# 2-Amino-4-*tert*-butyl-5-(4-chloro­benz­yl)thia­zol-3-ium chloride

**DOI:** 10.1107/S160053681000721X

**Published:** 2010-03-03

**Authors:** Jun-Mei Peng, Lin-Tao Yang, Zhi Qin, Ai-Xi Hu

**Affiliations:** a College of Chemistry and Chemical Engineering, Hunan University, Changsha 410082, People’s Republic of China

## Abstract

The title compound, C_14_H_18_ClN_2_S^+^·Cl^−^, crystallizes with two formula units in the asymmetric unit. The dihedral angles between the mean planes of the chloro­phenyl and thia­zole rings are 87.8 (2) and 88.0 (2)° in the two independent mol­ecules. In the crystal, the anions and cations are connected by N—H⋯Cl hydrogen bonds.

## Related literature

For 2-amino-4-aryl­thia­zol compounds, see Marcantonio *et al.* (2002 ▶) and for their synthesis, see: Hu *et al.* (2007 ▶). For related structures, see: Cao *et al.* (2007 ▶); He *et al.* (2006 ▶); Hu *et al.* (2007 ▶); Xu *et al.* (2007 ▶).
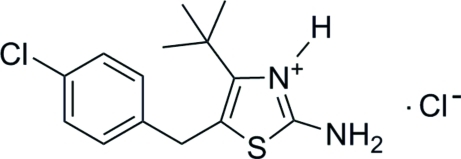


## Experimental

### 

#### Crystal data

C_14_H_18_ClN_2_S^+^·Cl^−^
*M* *_r_* = 317.26
Monoclinic, 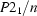

*a* = 12.0810 (5) Å
*b* = 17.0208 (8) Å
*c* = 16.6465 (7) Å
β = 108.587 (1)°
*V* = 3244.4 (2) Å^3^
*Z* = 8
Mo *K*α radiation
μ = 0.52 mm^−1^
*T* = 173 K
0.45 × 0.41 × 0.35 mm

#### Data collection

Bruker SMART 1000 CCD diffractometer
Absorption correction: multi-scan (*SADABS*; Sheldrick, 2004 ▶) *T*_min_ = 0.801, *T*_max_ = 0.840
16212 measured reflections
7029 independent reflections
5252 reflections with *I* > 2σ(*I*)
*R*_int_ = 0.021

#### Refinement

*R*[*F* ^2^ > 2σ(*F* ^2^)] = 0.038
*wR*(*F* ^2^) = 0.106
*S* = 1.03
7029 reflections
349 parameters
H-atom parameters constrained
Δρ_max_ = 0.37 e Å^−3^
Δρ_min_ = −0.19 e Å^−3^

### 

Data collection: *SMART* (Bruker, 2001 ▶); cell refinement: *SAINT-Plus* (Bruker, 2003 ▶); data reduction: *SAINT-Plus*; program(s) used to solve structure: *SHELXTL* (Sheldrick, 2008 ▶); program(s) used to refine structure: *SHELXTL*; molecular graphics: *SHELXTL*; software used to prepare material for publication: *SHELXTL*.

## Supplementary Material

Crystal structure: contains datablocks I, global. DOI: 10.1107/S160053681000721X/bt5189sup1.cif

Structure factors: contains datablocks I. DOI: 10.1107/S160053681000721X/bt5189Isup2.hkl

Additional supplementary materials:  crystallographic information; 3D view; checkCIF report

## Figures and Tables

**Table 1:** Hydrogen-bond geometry (Å, °)

*D*—H⋯*A*	*D*—H	H⋯*A*	*D*⋯*A*	*D*—H⋯*A*
N1—H1⋯Cl3	0.88	2.33	3.0882 (16)	144
N2—H2*A*⋯Cl3	0.88	2.34	3.1078 (19)	146
N2—H2*B*⋯Cl4	0.88	2.21	3.0327 (19)	155
N3—H3⋯Cl4	0.88	2.27	3.0289 (17)	145
N4—H4*A*⋯Cl4	0.88	2.36	3.1131 (19)	143
N4—H4*B*⋯Cl3^i^	0.88	2.22	3.0543 (19)	157

Symmetry code: (i) 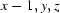.

**Fig. 1.**
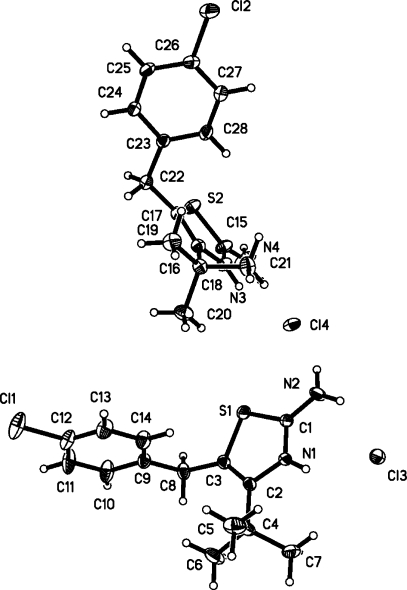
The structure of the title compound showing 50% probability displacement ellipsoids.

## supplementary crystallographic information

### Comment

Thiazole compounds are important   nitrogen-containing heterocyclic compounds, because of their wide range of biological activity. 2-Amino-4-arylthiazol compounds play an important role in the field of organic pharmaceutical chemistry (Marcantonio *et al.*, 2002). The synthesis of 2-amino-4-arylthiazoles was reported before (Cao *et al.*, 2007, He *et al.*, 2006, Hu *et al.*, 2007 b, Xu *et al.*, 2007). The title compound   was prepared as part of an ongoing investigation on the synthesis and structural properties of 2-amino-4-arylthiazole derivatives.

### Experimental

0.05 mol 2-Chloro-1-(4-chlorophenyl)-4,4-dimethyl-3-pentanone and 0.05 mol thiurea were dissolved in 100 ml EtOH and heated to reflux 12 h. After finishing the reaction, the solution was cooled and the precipitate formed was filtered out, dried, givingthe the title compound, yield 71.3 %. m.p.474–475.1 K.The crystals suitable for X-ray structure determination were obtained by slow evaporation of an ethanol solution at room temperation.

### Refinement

All H atoms were refined using a riding model, with N—H distances of 0.88 and C—H distances ranging from 0.95 to 0.98 Å, and with *U*_iso_(H) = 1.2U_eq_(C,N), or *U*_iso_(H) = 1.5U_eq_(C_methyl_).

### Crystal data

**Table:**

C_14_H_18_ClN_2_S^+^·Cl^−^	*F*(000) = 1328
*M**_r_* = 317.26	*D*_x_ = 1.299 Mg m^−^^3^
Monoclinic, *P*2_1_/*n*	Melting point: 474.5 K
Hall symbol: -P 2yn	Mo *K*α radiation, λ = 0.71073 Å
*a* = 12.0810 (5) Å	Cell parameters from 7242 reflections
*b* = 17.0208 (8) Å	θ = 2.2–27.0°
*c* = 16.6465 (7) Å	µ = 0.52 mm^−^^1^
β = 108.587 (1)°	*T* = 173 K
*V* = 3244.4 (2) Å^3^	Block, colorless
*Z* = 8	0.45 × 0.41 × 0.35 mm

### Data collection

**Table:**

Bruker SMART 1000 CCD diffractometer	7029 independent reflections
Radiation source: fine-focus sealed tube	5252 reflections with *I* > 2σ(*I*)
graphite	*R*_int_ = 0.021
ω scans	θ_max_ = 27.1°, θ_min_ = 1.8°
Absorption correction: multi-scan (*SADABS*; Sheldrick, 2004)	*h* = −15→15
*T*_min_ = 0.801, *T*_max_ = 0.840	*k* = −21→6
16212 measured reflections	*l* = −21→20

### Refinement

**Table:**

Refinement on *F*^2^	Primary atom site location: structure-invariant direct methods
Least-squares matrix: full	Secondary atom site location: difference Fourier map
*R*[*F*^2^ > 2σ(*F*^2^)] = 0.038	Hydrogen site location: inferred from neighbouring sites
*wR*(*F*^2^) = 0.106	H-atom parameters constrained
*S* = 1.03	*w* = 1/[σ^2^(*F*_o_^2^) + (0.0483*P*)^2^ + 1.4763*P*] where *P* = (*F*_o_^2^ + 2*F*_c_^2^)/3
7029 reflections	(Δ/σ)_max_ = 0.001
349 parameters	Δρ_max_ = 0.37 e Å^−^^3^
0 restraints	Δρ_min_ = −0.19 e Å^−^^3^

### Special details

**Table:**

Experimental. The ^1^H NMR (CDCl_3_,400 MHz) of 4-*tert*-butyl-5-(4-chlorobenzyl)thiazol-2-amine: δ (p.p.m.) 1.32(s, 9H, 3×CH_3_), 4.1(s, 2H, CH_2_),4.8(bs, 2H, NH_2_),7.12(d, *J* = 8.0 Hz, 2H, C_6_H_4_Cl 2,6-H),7.26(d, *J* = 8.0 Hz, 2H, C_6_H_4_Cl 3,5-H).
Geometry. All esds (except the esd in the dihedral angle between two l.s. planes) are estimated using the full covariance matrix. The cell esds are taken into account individually in the estimation of esds in distances, angles and torsion angles; correlations between esds in cell parameters are only used when they are defined by crystal symmetry. An approximate (isotropic) treatment of cell esds is used for estimating esds involving l.s. planes.
Refinement. Refinement of *F*^2^ against ALL reflections. The weighted *R*-factor wR and goodness of fit *S* are based on *F*^2^, conventional *R*-factors *R* are based on *F*, with *F* set to zero for negative *F*^2^. The threshold expression of *F*^2^ > σ(*F*^2^) is used only for calculating *R*-factors(gt) etc. and is not relevant to the choice of reflections for refinement. *R*-factors based on *F*^2^ are statistically about twice as large as those based on *F*, and *R*- factors based on ALL data will be even larger.

### Fractional atomic coordinates and isotropic or equivalent isotropic displacement parameters (Å^2^)

**Table:**

	*x*	*y*	*z*	*U*_iso_*/*U*_eq_	
Cl1	0.71220 (7)	0.00215 (5)	1.23595 (4)	0.0741 (2)	
Cl2	0.13936 (7)	0.72411 (4)	1.04620 (5)	0.06191 (19)	
Cl3	0.86628 (4)	0.27452 (3)	0.67478 (4)	0.03947 (13)	
Cl4	0.37086 (4)	0.25787 (3)	0.68564 (4)	0.04414 (14)	
S1	0.56742 (5)	0.11936 (3)	0.77844 (4)	0.03767 (14)	
S2	0.09432 (5)	0.31590 (4)	0.85845 (4)	0.04243 (15)	
C1	0.65841 (17)	0.17991 (11)	0.74588 (13)	0.0335 (4)	
C2	0.78072 (18)	0.07566 (11)	0.80669 (12)	0.0307 (4)	
C3	0.68127 (19)	0.05130 (11)	0.81964 (12)	0.0334 (4)	
C4	0.90343 (19)	0.04129 (12)	0.83044 (13)	0.0378 (5)	
C5	0.9824 (2)	0.08743 (17)	0.90556 (16)	0.0584 (7)	
H5A	0.9863	0.1423	0.8890	0.088*	
H5B	1.0610	0.0646	0.9231	0.088*	
H5C	0.9507	0.0850	0.9528	0.088*	
C6	0.9041 (3)	−0.04586 (15)	0.85414 (19)	0.0631 (7)	
H6A	0.8763	−0.0515	0.9031	0.095*	
H6B	0.9837	−0.0665	0.8684	0.095*	
H6C	0.8526	−0.0752	0.8060	0.095*	
C7	0.9498 (2)	0.04806 (17)	0.75503 (16)	0.0545 (7)	
H7A	0.8965	0.0209	0.7059	0.082*	
H7B	1.0274	0.0240	0.7698	0.082*	
H7C	0.9551	0.1036	0.7412	0.082*	
C8	0.6485 (2)	−0.01887 (12)	0.86164 (13)	0.0396 (5)	
H8A	0.6930	−0.0648	0.8521	0.048*	
H8B	0.5645	−0.0300	0.8335	0.048*	
C9	0.66972 (19)	−0.01103 (12)	0.95659 (13)	0.0346 (4)	
C10	0.6492 (2)	−0.07585 (14)	0.99990 (15)	0.0546 (7)	
H10	0.6256	−0.1239	0.9703	0.065*	
C11	0.6625 (3)	−0.07207 (16)	1.08559 (15)	0.0636 (8)	
H11	0.6478	−0.1170	1.1146	0.076*	
C12	0.6971 (2)	−0.00275 (15)	1.12798 (14)	0.0470 (6)	
C13	0.7210 (2)	0.06203 (14)	1.08781 (14)	0.0484 (6)	
H13	0.7467	0.1094	1.1182	0.058*	
C14	0.7073 (2)	0.05744 (13)	1.00207 (14)	0.0452 (5)	
H14	0.7240	0.1022	0.9739	0.054*	
C15	0.17384 (17)	0.28756 (13)	0.79473 (14)	0.0391 (5)	
C16	0.31809 (17)	0.33017 (11)	0.91593 (13)	0.0318 (4)	
C17	0.22313 (18)	0.34283 (12)	0.93980 (13)	0.0348 (4)	
C18	0.44803 (17)	0.34477 (12)	0.95687 (13)	0.0331 (4)	
C19	0.4745 (2)	0.38497 (14)	1.04315 (14)	0.0473 (6)	
H19A	0.4300	0.4340	1.0366	0.071*	
H19B	0.5582	0.3966	1.0658	0.071*	
H19C	0.4523	0.3501	1.0823	0.071*	
C20	0.51412 (19)	0.26639 (13)	0.96966 (16)	0.0462 (5)	
H20A	0.4873	0.2327	1.0075	0.069*	
H20B	0.5980	0.2762	0.9948	0.069*	
H20C	0.4992	0.2402	0.9148	0.069*	
C21	0.4904 (2)	0.39763 (14)	0.89765 (14)	0.0453 (5)	
H21A	0.4810	0.3699	0.8443	0.068*	
H21B	0.5729	0.4106	0.9248	0.068*	
H21C	0.4441	0.4461	0.8861	0.068*	
C22	0.2038 (2)	0.37428 (12)	1.01857 (13)	0.0383 (5)	
H22A	0.2725	0.3605	1.0677	0.046*	
H22B	0.1354	0.3470	1.0261	0.046*	
C23	0.18367 (16)	0.46231 (12)	1.02080 (12)	0.0314 (4)	
C24	0.15976 (19)	0.49386 (13)	1.09061 (13)	0.0409 (5)	
H24	0.1527	0.4596	1.1337	0.049*	
C25	0.1460 (2)	0.57353 (14)	1.09893 (14)	0.0449 (5)	
H25	0.1312	0.5940	1.1476	0.054*	
C26	0.15426 (18)	0.62310 (12)	1.03541 (14)	0.0388 (5)	
C27	0.17377 (19)	0.59376 (13)	0.96404 (13)	0.0395 (5)	
H27	0.1766	0.6281	0.9197	0.047*	
C28	0.18928 (19)	0.51355 (12)	0.95759 (13)	0.0374 (5)	
H28	0.2041	0.4933	0.9088	0.045*	
N1	0.76480 (14)	0.14883 (9)	0.76544 (10)	0.0305 (4)	
H1	0.8220	0.1728	0.7532	0.037*	
N2	0.62892 (16)	0.24695 (11)	0.70590 (14)	0.0518 (5)	
H2A	0.6808	0.2740	0.6905	0.062*	
H2B	0.5572	0.2649	0.6945	0.062*	
N3	0.28711 (14)	0.29818 (10)	0.83386 (11)	0.0340 (4)	
H3	0.3399	0.2859	0.8098	0.041*	
N4	0.13062 (16)	0.25857 (13)	0.71759 (13)	0.0569 (6)	
H4A	0.1777	0.2450	0.6891	0.068*	
H4B	0.0547	0.2528	0.6946	0.068*	

### Atomic displacement parameters (Å^2^)

**Table:**

	*U*^11^	*U*^22^	*U*^33^	*U*^12^	*U*^13^	*U*^23^
Cl1	0.0859 (5)	0.1070 (6)	0.0370 (3)	−0.0352 (4)	0.0301 (3)	−0.0158 (3)
Cl2	0.0842 (5)	0.0404 (3)	0.0755 (5)	0.0100 (3)	0.0456 (4)	−0.0052 (3)
Cl3	0.0308 (3)	0.0335 (3)	0.0563 (3)	−0.0032 (2)	0.0168 (2)	0.0068 (2)
Cl4	0.0338 (3)	0.0539 (3)	0.0475 (3)	0.0088 (2)	0.0170 (2)	−0.0081 (2)
S1	0.0384 (3)	0.0332 (3)	0.0488 (3)	−0.0005 (2)	0.0242 (2)	0.0046 (2)
S2	0.0306 (3)	0.0534 (3)	0.0479 (3)	−0.0055 (2)	0.0191 (2)	−0.0129 (3)
C1	0.0339 (11)	0.0289 (10)	0.0426 (12)	0.0028 (8)	0.0191 (9)	0.0053 (8)
C2	0.0418 (11)	0.0245 (9)	0.0255 (10)	0.0034 (8)	0.0102 (8)	−0.0001 (7)
C3	0.0459 (12)	0.0245 (9)	0.0315 (10)	0.0014 (8)	0.0147 (9)	0.0005 (8)
C4	0.0428 (12)	0.0316 (11)	0.0364 (11)	0.0107 (9)	0.0090 (9)	0.0027 (9)
C5	0.0449 (14)	0.0663 (17)	0.0520 (15)	0.0113 (12)	−0.0015 (12)	−0.0127 (13)
C6	0.0666 (17)	0.0408 (14)	0.0777 (19)	0.0192 (13)	0.0172 (15)	0.0162 (13)
C7	0.0453 (13)	0.0696 (17)	0.0521 (15)	0.0258 (13)	0.0205 (11)	0.0077 (13)
C8	0.0595 (14)	0.0280 (10)	0.0332 (11)	−0.0068 (9)	0.0172 (10)	0.0009 (8)
C9	0.0425 (12)	0.0307 (10)	0.0338 (11)	−0.0053 (9)	0.0164 (9)	−0.0002 (8)
C10	0.0857 (19)	0.0417 (13)	0.0420 (13)	−0.0294 (13)	0.0284 (13)	−0.0065 (10)
C11	0.100 (2)	0.0567 (16)	0.0399 (14)	−0.0356 (15)	0.0311 (14)	−0.0010 (12)
C12	0.0496 (13)	0.0640 (15)	0.0326 (12)	−0.0179 (12)	0.0206 (10)	−0.0089 (10)
C13	0.0587 (15)	0.0484 (13)	0.0407 (13)	−0.0160 (12)	0.0195 (11)	−0.0134 (10)
C14	0.0623 (15)	0.0334 (11)	0.0447 (13)	−0.0125 (10)	0.0240 (11)	−0.0034 (10)
C15	0.0281 (10)	0.0450 (12)	0.0471 (13)	−0.0062 (9)	0.0159 (9)	−0.0145 (10)
C16	0.0342 (10)	0.0256 (9)	0.0361 (11)	−0.0035 (8)	0.0117 (9)	−0.0034 (8)
C17	0.0374 (11)	0.0328 (10)	0.0358 (11)	−0.0027 (9)	0.0139 (9)	−0.0025 (8)
C18	0.0331 (10)	0.0292 (10)	0.0342 (11)	−0.0053 (8)	0.0067 (8)	−0.0019 (8)
C19	0.0455 (13)	0.0499 (14)	0.0411 (13)	−0.0052 (11)	0.0064 (10)	−0.0121 (10)
C20	0.0384 (12)	0.0364 (12)	0.0591 (15)	0.0005 (9)	0.0089 (11)	−0.0026 (10)
C21	0.0401 (12)	0.0470 (13)	0.0447 (13)	−0.0166 (10)	0.0078 (10)	0.0030 (10)
C22	0.0460 (12)	0.0386 (11)	0.0354 (11)	0.0022 (9)	0.0199 (10)	0.0022 (9)
C23	0.0270 (10)	0.0395 (11)	0.0275 (10)	0.0039 (8)	0.0085 (8)	0.0000 (8)
C24	0.0469 (13)	0.0484 (13)	0.0327 (11)	0.0143 (10)	0.0203 (10)	0.0085 (9)
C25	0.0519 (14)	0.0530 (14)	0.0365 (12)	0.0184 (11)	0.0236 (10)	0.0014 (10)
C26	0.0368 (11)	0.0366 (11)	0.0452 (12)	0.0068 (9)	0.0161 (10)	−0.0027 (9)
C27	0.0449 (12)	0.0402 (12)	0.0376 (12)	0.0008 (10)	0.0191 (10)	0.0037 (9)
C28	0.0480 (13)	0.0402 (11)	0.0274 (10)	−0.0005 (9)	0.0169 (9)	−0.0030 (8)
N1	0.0308 (8)	0.0263 (8)	0.0377 (9)	0.0031 (7)	0.0154 (7)	0.0052 (7)
N2	0.0365 (10)	0.0377 (10)	0.0897 (16)	0.0130 (8)	0.0322 (10)	0.0280 (10)
N3	0.0271 (8)	0.0378 (9)	0.0394 (10)	−0.0060 (7)	0.0135 (7)	−0.0120 (7)
N4	0.0279 (9)	0.0891 (16)	0.0538 (12)	−0.0124 (10)	0.0129 (9)	−0.0382 (12)

### Geometric parameters (Å, °)

**Table:**

Cl1—C12	1.750 (2)	C15—N4	1.318 (3)
Cl2—C26	1.744 (2)	C15—N3	1.327 (3)
S1—C1	1.716 (2)	C16—C17	1.346 (3)
S1—C3	1.762 (2)	C16—N3	1.406 (2)
S2—C15	1.712 (2)	C16—C18	1.519 (3)
S2—C17	1.767 (2)	C17—C22	1.502 (3)
C1—N2	1.312 (3)	C18—C19	1.530 (3)
C1—N1	1.331 (2)	C18—C20	1.534 (3)
C2—C3	1.352 (3)	C18—C21	1.537 (3)
C2—N1	1.406 (2)	C19—H19A	0.9800
C2—C4	1.524 (3)	C19—H19B	0.9800
C3—C8	1.500 (3)	C19—H19C	0.9800
C4—C5	1.527 (3)	C20—H20A	0.9800
C4—C7	1.533 (3)	C20—H20B	0.9800
C4—C6	1.534 (3)	C20—H20C	0.9800
C5—H5A	0.9800	C21—H21A	0.9800
C5—H5B	0.9800	C21—H21B	0.9800
C5—H5C	0.9800	C21—H21C	0.9800
C6—H6A	0.9800	C22—C23	1.520 (3)
C6—H6B	0.9800	C22—H22A	0.9900
C6—H6C	0.9800	C22—H22B	0.9900
C7—H7A	0.9800	C23—C28	1.385 (3)
C7—H7B	0.9800	C23—C24	1.392 (3)
C7—H7C	0.9800	C24—C25	1.378 (3)
C8—C9	1.524 (3)	C24—H24	0.9500
C8—H8A	0.9900	C25—C26	1.381 (3)
C8—H8B	0.9900	C25—H25	0.9500
C9—C10	1.383 (3)	C26—C27	1.377 (3)
C9—C14	1.385 (3)	C27—C28	1.387 (3)
C10—C11	1.385 (3)	C27—H27	0.9500
C10—H10	0.9500	C28—H28	0.9500
C11—C12	1.370 (3)	N1—H1	0.8800
C11—H11	0.9500	N2—H2A	0.8800
C12—C13	1.368 (3)	N2—H2B	0.8800
C13—C14	1.385 (3)	N3—H3	0.8800
C13—H13	0.9500	N4—H4A	0.8800
C14—H14	0.9500	N4—H4B	0.8800
			
C1—S1—C3	91.06 (10)	N3—C16—C18	114.59 (17)
C15—S2—C17	90.97 (10)	C16—C17—C22	134.4 (2)
N2—C1—N1	123.71 (18)	C16—C17—S2	110.96 (15)
N2—C1—S1	125.80 (16)	C22—C17—S2	114.66 (15)
N1—C1—S1	110.49 (14)	C16—C18—C19	111.79 (17)
C3—C2—N1	111.08 (17)	C16—C18—C20	109.72 (16)
C3—C2—C4	132.96 (18)	C19—C18—C20	108.34 (18)
N1—C2—C4	115.92 (17)	C16—C18—C21	108.38 (16)
C2—C3—C8	134.36 (19)	C19—C18—C21	109.12 (17)
C2—C3—S1	111.08 (14)	C20—C18—C21	109.46 (18)
C8—C3—S1	114.55 (16)	C18—C19—H19A	109.5
C2—C4—C5	108.51 (17)	C18—C19—H19B	109.5
C2—C4—C7	109.74 (16)	H19A—C19—H19B	109.5
C5—C4—C7	109.6 (2)	C18—C19—H19C	109.5
C2—C4—C6	111.26 (19)	H19A—C19—H19C	109.5
C5—C4—C6	109.6 (2)	H19B—C19—H19C	109.5
C7—C4—C6	108.1 (2)	C18—C20—H20A	109.5
C4—C5—H5A	109.5	C18—C20—H20B	109.5
C4—C5—H5B	109.5	H20A—C20—H20B	109.5
H5A—C5—H5B	109.5	C18—C20—H20C	109.5
C4—C5—H5C	109.5	H20A—C20—H20C	109.5
H5A—C5—H5C	109.5	H20B—C20—H20C	109.5
H5B—C5—H5C	109.5	C18—C21—H21A	109.5
C4—C6—H6A	109.5	C18—C21—H21B	109.5
C4—C6—H6B	109.5	H21A—C21—H21B	109.5
H6A—C6—H6B	109.5	C18—C21—H21C	109.5
C4—C6—H6C	109.5	H21A—C21—H21C	109.5
H6A—C6—H6C	109.5	H21B—C21—H21C	109.5
H6B—C6—H6C	109.5	C17—C22—C23	116.30 (17)
C4—C7—H7A	109.5	C17—C22—H22A	108.2
C4—C7—H7B	109.5	C23—C22—H22A	108.2
H7A—C7—H7B	109.5	C17—C22—H22B	108.2
C4—C7—H7C	109.5	C23—C22—H22B	108.2
H7A—C7—H7C	109.5	H22A—C22—H22B	107.4
H7B—C7—H7C	109.5	C28—C23—C24	117.70 (19)
C3—C8—C9	115.56 (17)	C28—C23—C22	123.74 (18)
C3—C8—H8A	108.4	C24—C23—C22	118.56 (18)
C9—C8—H8A	108.4	C25—C24—C23	121.8 (2)
C3—C8—H8B	108.4	C25—C24—H24	119.1
C9—C8—H8B	108.4	C23—C24—H24	119.1
H8A—C8—H8B	107.5	C24—C25—C26	118.94 (19)
C10—C9—C14	117.9 (2)	C24—C25—H25	120.5
C10—C9—C8	118.15 (18)	C26—C25—H25	120.5
C14—C9—C8	123.92 (18)	C27—C26—C25	120.9 (2)
C9—C10—C11	121.2 (2)	C27—C26—Cl2	119.87 (17)
C9—C10—H10	119.4	C25—C26—Cl2	119.25 (16)
C11—C10—H10	119.4	C26—C27—C28	119.2 (2)
C12—C11—C10	119.1 (2)	C26—C27—H27	120.4
C12—C11—H11	120.4	C28—C27—H27	120.4
C10—C11—H11	120.4	C23—C28—C27	121.38 (19)
C13—C12—C11	121.3 (2)	C23—C28—H28	119.3
C13—C12—Cl1	119.65 (18)	C27—C28—H28	119.3
C11—C12—Cl1	119.02 (19)	C1—N1—C2	116.29 (16)
C12—C13—C14	118.9 (2)	C1—N1—H1	121.9
C12—C13—H13	120.5	C2—N1—H1	121.9
C14—C13—H13	120.5	C1—N2—H2A	120.0
C9—C14—C13	121.4 (2)	C1—N2—H2B	120.0
C9—C14—H14	119.3	H2A—N2—H2B	120.0
C13—C14—H14	119.3	C15—N3—C16	116.31 (17)
N4—C15—N3	123.75 (19)	C15—N3—H3	121.8
N4—C15—S2	125.65 (16)	C16—N3—H3	121.8
N3—C15—S2	110.60 (15)	C15—N4—H4A	120.0
C17—C16—N3	111.16 (17)	C15—N4—H4B	120.0
C17—C16—C18	134.23 (18)	H4A—N4—H4B	120.0
			
C3—S1—C1—N2	179.1 (2)	N3—C16—C17—S2	−0.9 (2)
C3—S1—C1—N1	0.27 (16)	C18—C16—C17—S2	177.18 (19)
N1—C2—C3—C8	−177.9 (2)	C15—S2—C17—C16	0.42 (17)
C4—C2—C3—C8	−0.4 (4)	C15—S2—C17—C22	−179.73 (17)
N1—C2—C3—S1	1.0 (2)	C17—C16—C18—C19	−2.1 (3)
C4—C2—C3—S1	178.50 (18)	N3—C16—C18—C19	175.88 (18)
C1—S1—C3—C2	−0.75 (16)	C17—C16—C18—C20	118.1 (3)
C1—S1—C3—C8	178.39 (16)	N3—C16—C18—C20	−63.9 (2)
C3—C2—C4—C5	−103.9 (3)	C17—C16—C18—C21	−122.4 (3)
N1—C2—C4—C5	73.5 (2)	N3—C16—C18—C21	55.6 (2)
C3—C2—C4—C7	136.4 (2)	C16—C17—C22—C23	91.1 (3)
N1—C2—C4—C7	−46.2 (2)	S2—C17—C22—C23	−88.7 (2)
C3—C2—C4—C6	16.8 (3)	C17—C22—C23—C28	−3.9 (3)
N1—C2—C4—C6	−165.77 (19)	C17—C22—C23—C24	176.98 (19)
C2—C3—C8—C9	87.5 (3)	C28—C23—C24—C25	−2.3 (3)
S1—C3—C8—C9	−91.4 (2)	C22—C23—C24—C25	176.9 (2)
C3—C8—C9—C10	−175.3 (2)	C23—C24—C25—C26	1.2 (4)
C3—C8—C9—C14	5.3 (3)	C24—C25—C26—C27	1.1 (3)
C14—C9—C10—C11	1.8 (4)	C24—C25—C26—Cl2	−178.98 (18)
C8—C9—C10—C11	−177.6 (3)	C25—C26—C27—C28	−2.2 (3)
C9—C10—C11—C12	−0.3 (5)	Cl2—C26—C27—C28	177.89 (17)
C10—C11—C12—C13	−1.4 (5)	C24—C23—C28—C27	1.1 (3)
C10—C11—C12—Cl1	179.3 (2)	C22—C23—C28—C27	−178.0 (2)
C11—C12—C13—C14	1.5 (4)	C26—C27—C28—C23	1.1 (3)
Cl1—C12—C13—C14	−179.3 (2)	N2—C1—N1—C2	−178.6 (2)
C10—C9—C14—C13	−1.8 (4)	S1—C1—N1—C2	0.3 (2)
C8—C9—C14—C13	177.6 (2)	C3—C2—N1—C1	−0.9 (2)
C12—C13—C14—C9	0.2 (4)	C4—C2—N1—C1	−178.81 (17)
C17—S2—C15—N4	179.8 (2)	N4—C15—N3—C16	179.7 (2)
C17—S2—C15—N3	0.19 (17)	S2—C15—N3—C16	−0.8 (2)
N3—C16—C17—C22	179.3 (2)	C17—C16—N3—C15	1.1 (3)
C18—C16—C17—C22	−2.6 (4)	C18—C16—N3—C15	−177.38 (18)

### Hydrogen-bond geometry (Å, °)

**Table:**

*D*—H···*A*	*D*—H	H···*A*	*D*···*A*	*D*—H···*A*
N1—H1···Cl3	0.88	2.33	3.0882 (16)	144
N2—H2A···Cl3	0.88	2.34	3.1078 (19)	146
N2—H2B···Cl4	0.88	2.21	3.0327 (19)	155
N3—H3···Cl4	0.88	2.27	3.0289 (17)	145
N4—H4A···Cl4	0.88	2.36	3.1131 (19)	143
N4—H4B···Cl3^i^	0.88	2.22	3.0543 (19)	157

Symmetry codes: (i) *x*−1, *y*, *z*.
